# Systemic vascular function, measured with forearm flow mediated dilatation, in acute and stable cerebrovascular disease: a case-control study

**DOI:** 10.1186/1476-7120-8-46

**Published:** 2010-10-19

**Authors:** Christopher D Beer, Kathleen Potter, David Blacker, Leonard Arnolda, Graeme J Hankey, Ian B Puddey

**Affiliations:** 1 Western Australian Centre for Health and Ageing, University of Western Australia, Perth, Australia; 2 School of Medicine and Pharmacology, University of Western Australia, Perth, Australia; 3 Centre for Medical Research, Western Australian Institute for Medical Research, Perth, Australia; 4 Stroke Unit, Royal Perth Hospital, Perth, Australia; 5 Stroke Unit, Sir Charles Gairdner Hospital, Perth, Australia; 6 Cardiology, The Canberra Hospital, Canberra, Australia; 7 Medical School, Australian National University, Canberra, Australia

## Abstract

**Background:**

Acute ischaemic stroke is associated with alteration in systemic markers of vascular function. We measured forearm vascular function (using forearm flow mediated dilatation) to clarify whether recent acute ischaemic stroke/TIA is associated with impaired systemic vascular function.

**Methods:**

Prospective case control study enrolling 17 patients with recent acute ischaemic stroke/TIA and 17 sex matched controls with stroke more than two years previously. Forearm vascular function was measured using flow medicated dilatation (FMD).

**Results:**

Flow mediated dilatation was 6.0 ± 1.1% in acute stroke/TIA patients and 4.7 ± 1.0% among control subjects (p = 0.18). The mean paired difference in FMD between subjects with recent acute stroke and controls was 1.25% (95% CI -0.65, 3.14; p = 0.18). Endothelium independent dilatation was measured in six pairs of participants and was similar in acute stroke/TIA patients (22.6 ± 4.3%) and control subjects (19.1 ± 2.6%; p = 0.43).

**Conclusions:**

Despite the small size of this study, these data indicate that recent acute stroke is not necessarily associated with a clinically important reduction in FMD.

## Introduction

Endothelial dysfunction, as measured by impaired brachial flow-mediated dilatation, occurs early in atherosclerosis. It is associated with subsequent intracranial small vessel disease (as manifest by small, deep, lacunar ischaemic stroke and brain imaging evidence of deep white matter infarction), and is an independent predictor of vascular events[1-4]. Moreover, endothelial dysfunction can be reversed with treatments that lower C-reactive protein and homocysteine, suggesting that systemic inflammation may mediate the observed vascular dysfunction in patients with stable cerebrovascular disease[5].

However, the importance of endothelial dysfunction in the pathogenesis of acute ischaemic stroke and transient ischaemic attacks (TIA) remains uncertain. Acute ischaemic stroke is associated with a rise in markers of endothelial activation in the systemic circulation [6,7] and with fewer circulating endothelial progenitor cells [8] but whether these results reflect an underlying primary disturbance of endothelial function in the pathogenesis of acute ischaemic stroke, or an acute phase response to the cerebral injury, is unknown. The endothelial marker E-selectin is elevated in acute stroke and returns to normal concentrations within 3-6 months[6]. However it is not known if endothelial function is impaired in subjects with acute stroke (compared to those who have suffered a remote stroke).

We hypothesised that acute ischaemic stroke would be associated with impairment of systemic endothelial dysfunction. Our aim was to measure systemic vascular function directly (using forearm flow mediated dilatation) in patients with acute ischaemic stroke, and controls with stable cerebrovascular disease, to clarify whether recent acute ischaemic stroke/TIA is in fact associated with impaired systemic vascular function.

## Materials and methods

### Ethics

The research was approved by the Royal Perth Hospital Ethics Committee. All participants provided written consent.

### Design

Prospective case-control study.

### Setting

Acute stroke units and TIA clinics of two university teaching hospitals in Perth, Australia.

### Subjects

Cases of recent acute TIA or ischaemic stroke

#### Inclusion criteria

Patients with a clinical diagnosis of acute ischaemic stroke or TIA within the previous 10 days. All participants underwent brain imaging with computed tomography. Clinical records were reviewed subsequent to the patient's discharge to confirm a final clinical diagnosis of an acute cerebral ischaemic event and classify the clinical syndrome using the Oxfordshire Community Stroke Project classification [9].

#### Exclusion criteria

Patients with blood glucose level >13 mmol/L and those with diagnosed infection or malignancy.

#### Controls

Control participants were patients who had a history of acute ischaemic stroke or TIA more than two years previously, had been enrolled in the VITamins TO Prevent Stroke Trial,[10] taken part in a completed sub-study of arterial inflammation,[11] and were placebo treated. Sex matched control subjects closest to the age of cases with acute stroke/TIA patients were selected. This resulted in sex matched controls with mean age difference of ± 1.1 years (maximum ± 7 years) from recent acute stroke/TIA subjects at the time of assessment of forearm flow mediated dilatation.

### Baseline measures

Baseline variables, measured upon presentation to hospital at the time of the qualifying cerebrovascular event, for both cases and controls included demographic and clinical features, and are shown in table 1. Table 1 also shows age of the cases and control participants at the time of assessment of forearm flow mediated dilatation.

**Table 1:** Clinical variables

	Recent Stroke/TIA n = 17	Stable Disease n = 17	p
Gender, n = F	6 (35%)	6 (35%)	
Age at FMD scan, years	64.2 ± 13.0	64.7 ± 11.7	0.23
**Medical History**			
Myocardial Infarction	2 (12%)	1 (6%)	1.0
Hypertension	15 (89%)	15 (89%)	1.0
Hypercholesterolaemia	11 (65%)	12 (71%)	0.71
Diabetes	2 (12%)	3 (18%)	1.0
Smoking	9 (53%)	9 (53%)	1.0
**Prior Therapy**			
Antihypertensive	12 (71%)	14 (83%)	0.6
Lipid Lowering	12 (71%)	11 (65%)	0.72
**Clinical Variables**			
Clinical Syndrome TACS	1 (6%)	2 (12%)	
PACS	6 (35%)	4 (24%)	
LACS	4 (24%)	6 (35%)	
POCS	2 (12%)	1 (6%)	
TIA	4 (24%)	4 (24%)	
SBP, mmHg	139.9 ± 21.0	138.9 ± 18.7	0.86
DBP, mm Hg	84.1 ± 13.6	79.6 ± 14.7	0.21
Cholesterol, mmolL^-1^	3.9 ± 0.9	4.2 ± 0.9	0.44
Triglycerides,	1.1 ± 0.3	1.3 ± 0.5	0.25
HDL, mmolL^-1^	2.1 ± 1.1	2.2 ± 0.6	0.77
LDL, mmolL^-1^	1.3 ± 0.5	1.4 ± 0.6	0.52

Data are N (%) or N ± SD; TACI = Total Anterior Circulation, PACI = Partial Anterior Circulation, LACI = Lacunar, TIA = Transient Ischaemic attack; S(D)BP = Systolic (Diastolic) Blood Pressure; H(L)DL = High (Low) Density Lipoprotein

### Outcome Measures

The primary outcome was forearm flow mediated dilatation (FMD). FMD was measured by a single operator using a semi-automated custom-designed edge-detection and wall-tracking software designed to reduce investigator bias, as described previously [12]. Video files are provided demonstrating set-up (Additional File 1) and acquisition of images (Additional File 2). Briefly, participants lay supine on a flat examination couch in a quiet laboratory and B-mode ultrasound images were recorded using a 10 MHz multi-frequency linear array probe attached to a high-resolution ultrasound (Acuson Aspen, Mountain View, California). FMD was measured in the left arm except in subjects with a left hemiplegia. B-mode images of the brachial artery were obtained and recorded continuously for one minute to assess baseline arterial diameter. A rapid inflation/deflation pneumatic cuff placed around the forearm was inflated to 250 mmHg for five minutes. Post cuff deflation images were recorded continuously for two minutes to capture peak arterial dilation. Glyceryl trinitrate (GTN) mediated dilatation was also measured for the first six participants. In light of the high prevalence of multi-agent antihypertensive therapy it was considered prudent to complete data collection without exposing additional participants to GTN (which can be associated with systemic hypotension). FMD and GTN mediated dilation were measured off-line. The mean intra-observer coefficient of variation for FMD and GTN-mediated dilation measured by this technique are 6.7% and 3.9% respectively [12].

### Statistical Analysis

Log-normal data were log transformed prior to analysis and compared using paired t tests. Non-normal data were analysed using non parametric tests. Categorical variables were assessed with the chi squared or fisher exact test. Data were analysed using SPSS 15.0

## Results

### Baseline measures

We recruited 17 patients with recent acute ischaemic stroke (n = 16) or TIA (n = 1), and 17 age- and sex-matched controls who had experienced an ischaemic stroke or TIA more than two (median 4) years previously.

Cases and controls were adequately matched, with no significant differences between the two groups in the variables evaluated. (Table 1) The presenting syndromes of the cases with recent acute ischaemic stroke, and control participants, were varied. Among the patients with recent acute stroke or TIA, 1 had a total anterior circulation syndrome, 6 had partial anterior circulation syndromes, 4 had lacunar syndromes, 2 had posterior circulation syndromes, and 4 had presented with a TIA. Control subjects had a history of TIA (n = 4) or total anterior (n = 2), partial anterior (n = 4), lacunar (n = 6) or posterior (n = 1) syndrome ischaemic stroke.

### Outcome measures

Table 2 shows that the baseline arterial diameter, flow mediated dilatation and endothelium independent dilatation were similar in participants with recent acute stroke/TIA and controls with stable cerebrovascular disease. The mean paired difference in FMD between subjects with recent acute stroke and controls was 1.25% (95% CI -0.65, 3.14; p = 0.18).

**Table 2:** Forearm vascular function.

	Acute Stroke or TIA Mean ± SEM	Stable Cerebrovascular Disease Mean ± SEM	p
*Endothelium dependent vasodilatation*	*n = 17*	*n = 17*	
Baseline Diameter (mm)	3.6 ± 0.2	3.5 ± 0.2	0.53
FMD (%)	6.0 ± 1.1	4.7 ± 1.0	0.18

*Endothelium independent vasodilatation*	*n = 6*	*n = 6*	
GTN-mediated dilatation (%)	22.6 ± 4.3	19.1 ± 2.6	0.43

Mean blood pressure after the administration of GTN to 6 subjects with recent acute stroke/TIA was 144.4/85.6 mmHg, which did not differ significantly from the baseline mean systolic (152.2 mmHg; p = 0.34) or diastolic (87.2 mmHg, p = 0.83) blood pressures among this group. No adverse events related to the administration of GTN were observed.

## Discussion

### Principal findings

These data suggest that vascular function, measured with FMD, is similar in patients with recent acute and stable cerebrovascular disease. If anything, FMD tended to be better in subjects with acute stroke, which was an unexpected finding. These data do not support the hypothesis that acute stroke is associated with worsening of systemic vascular function. The lower limit of the 95% confidence interval of the mean paired differences between subjects with recent acute stroke and controls with prior cerebrovascular disease was -0.65%. This effectively excludes a worsening of FMD associated with acute ischaemic stroke of a magnitude which would be considered clinically important (>2%)[13].

Currently, concern regarding the safety of administration of GTN to people with acute ischaemic stroke precludes full measurement of FMD in acute stroke patients outside research protocols. Our preliminary data suggest that GTN mediated FMD can be safely assessed in people with recent acute ischaemic stroke. Further work in this population would ideally assess flow and GTN mediated dilatation, to ensure any observed differences are endothelium dependent (rather than due to differences in smooth muscle function, for example related to statin therapy). Safety of assessment of FMD in the acute phase of stroke could be confirmed by continuous BP monitoring whilst GTN is administered, combined with assessment of cerebral perfusion.

### Results in context of other studies

Our findings are consistent with other data suggesting that endothelial function is not necessarily worse in subjects with recent acute stroke compared to subjects with stable disease. For example, individuals with symptomatic ICA stenosis and severe periventricular white matter lesions had higher levels of VCAM-1 (a marker of endothelial cell activation) than subjects with acute stroke[14]. We are aware of only one other study that has assessed FMD in patients with acute stroke[15]. Those data were collected to test the hypothesis that endothelial dysfunction contributes to the risk of arterial stroke to a greater extent than embolic stroke, and thus did not utilize healthy or disease controls. Patients hospitalised with ischaemic stroke and atrial fibrillation within 5-10 days (n = 20) were found to have less impaired vascular function (5.7 ± 3.3%) than patients with ischaemic stroke in sinus rhythm (n = 32; 3.2 ± 2.4%; p = 0.041)[15]. Future work should stratify participants according to stroke sub-type. FMD has been found to be associated with survival free from cardiac events,[16] and composite cardiovascular outcomes[4]. Although our data suggest that FMD does not differ significantly between patients with acute stroke and stable cerebrovascular disease, future work should determine whether the degree of impairment of FMD is associated with future cerebrovascular events.

### Strengths of our study

To our knowledge this is the first study comparing forearm vascular function in people with recent acute ischaemic stroke or TIA and controls with stable cerebrovascular disease. Inclusion of a control population, sharing similar risk factor and treatment profiles to the cases with acute ischaemic stroke/TIA, is a strength of the study. Our aim was to determine the association of *acute *stroke with vascular function, and we thus chose to control for background cerebrovascular disease and treatments. However the study would have been furthered strengthened by the simultaneous inclusion of a healthy control population. Our methodology is also likely to be reliable, as the measurements were made by a single observer using semi-automated edge detection software. Forearm flow mediated dilatation (FMD) is a validated non invasive marker of systemic endothelial function and a functional assay of endothelium-derived nitric oxide.

### Potential weaknesses

The major weakness of our study is the potential for random error because of the small number of patients and controls studied. Because of the small data set, the results may be falsely negative. Further, there is potential for selection bias as the cases and controls were selected from hospital and clinical trial populations respectively. Interpretation of our data is limited by the heterogenous nature of the participants with acute stroke/TIA. It might be expected, for example, that participants with lacunar or embolic stroke are less likely to show evidence of vascular dysfunction than participants with large artery embolic stroke. Our participants were heavily pre-treated which may influence the observed results, given that commonly used medications, such as statins, may improve vascular function and reduce the risk of stroke[17,18]. We also enrolled patients up to 10 days after symptom onset and thus may have failed to ascertain differences in the very early phase of acute stroke. Future work should enrol patients in the very early phase after stroke (ideally within first 24 hours of ictus).

## Conclusion

• Despite the small size of this study, these data indicate that recent acute stroke may not necessarily be associated with a clinically important reduction in FMD.

• These data suggest that subjects with stroke more than two years previously have similar vascular function to subjects with recent stroke. The clinical utility of assessment of FMD in subjects with recent acute ischaemic stroke thus remains uncertain.

## Competing interests

The authors declare that they have no competing interests.

## Authors' contributions

CB designed the study, carried out or supervised subject recruitment and collection of data, analysed the data and wrote the manuscript. KP carried out assessment of flow mediated dilatation and assisted in recruitment, collection of data and provision of control data. DB assisted recruitment and data collection at the Sir Charles Gairdner site and assisted data interpretation and manuscript revision. LA, IBP and GH assisted in study design, data interpretation and manuscript revision. All authors read and approved the final manuscript.

## Supplementary Material

Additional file 1 **Set-up of semi-automated software for measurement of flow mediated dilatation**. A video file, demonstrating preparation of the semi-automated system Click here for file

Additional file 2 **Acquisition of flow mediated dilatation data using semi-automated software**. A sample video file, demonstrating acquisition of flow mediated dilatation data Click here for file
